# Global and regional cardiac magnetic resonance feature tracking left ventricular strain analysis in assessing early myocardial disease in β thalassemia major patients

**DOI:** 10.1186/s44348-024-00026-1

**Published:** 2024-08-03

**Authors:** Nihal M. Batouty, Ahmad M. Tawfik, Donia M. Sobh, Basma N. Gadelhak, Shimaa El-Ashwah, Mohamed Abdelghafar Hussein, Mai Gad, A. Ashraf Abd El Aziz, Mahmoud Abd El-Shahed, Rasha Karam

**Affiliations:** 1 Department of Diagnostic Radiology, Faculty of Medicine, Mansoura University, Elgomhoria St., Mansoura, 35516 Egypt; 2 Department of Hematology, Faculty of Medicine, Mansoura University, Elgomhoria St., Mansoura, 35516 Egypt; 3 Department of Pediatrics, Faculty of Medicine, Kafrelsheikh University, Kafr El Sheikh, Egypt; 4 Faculty of Medicine, Student Hospital, Mansoura University, Mansoura, Egypt

**Keywords:** Thalassemia, Sprains and strains, Myocardium magnetic resonance imaging, Iron overload

## Abstract

**Background:**

Cardiac magnetic resonance imaging (CMR) is the modality of choice for quantification of myocardial iron overload in β-thalassemia major patients using the T2* sequence. CMR feature tracking (FT) is a recent magnetic resonance imaging tool that gives an idea about myocardial fibers deformation; thus, it can detect early impairment in myocardial function even before the reduction in ejection fraction.

**Methods:**

This study aims to assess the ability of left ventricular CMR-FT in the early detection of systolic dysfunction in β thalassemia major patients and to correlate it with the degree of myocardial iron overload measured by CMR T2*. This prospective study enrolled 57 β thalassemia major patients who received long-term blood transfusion and 20 healthy controls. CMR was used to evaluate left ventricular volumes, ejection fraction, and the amount of myocardial T2*. A two-dimensional left ventricular FT analysis was performed. Both global and segmental left ventricular strain values were obtained.

**Results:**

The mean global circumferential strain (GCS) and global radial strain (GRS) values were significantly lower in patients compared to control (*P* = 0.002 and *P* = 0.006, respectively). No correlation was found between T2* values and ejection fraction; however, there was a significant correlation between T2* values and GCS and GRS (*P* = 0.012 and *P* = 0.025, respectively) in thalassemia patients. Regional strain revealed significantly lower values of GCS and GRS in basal regions compared to apical ones (*P* = 0.000).

**Conclusions:**

Our study revealed that CMR-FT can play a role in the early detection of systolic impairment in thalassemia patients.

## Background

Β Thalassemia major is a group of hereditary disorders causing chronic hemolytic anemia with the affected children requiring blood transfusions during their whole life. Lifelong blood transfusions cause increased iron absorption subsequently leading to myocardial iron overload (MIO) which is not completely reversible and represents the major cause of cardiac functional impairment and is the main etiology for morbidity and mortality [1, 2].

Early adequate treatment with iron chelation therapy before the development of heart failure can reverse MIO-induced cardiomyopathy, hence the need for its early detection [1]. β Thalassemia major patients receive iron chelation therapy after about 20 to 25 units of red blood cells transfusion. The cutoff value for the start of iron chelation therapy is a serum ferritin level of more than 1,000 ng/mL and hepatic iron concentration of more than 3 mg/gm measured either by liver biopsy or noninvasive cardiac magnetic resonance imaging (CMR) [3].

CMR is the modality of choice for quantification of MIO in thalassemia patients using noninvasive T2* sequence [4, 5]. CMR is the gold standard modality for the evaluation of ventricular function and early prediction of cardiac complications in thalassemia patients [2].

Many studies evaluated the role of left ventricular myocardial strain derived from echocardiography for early detection of impaired myocardial function in thalassemia patients [6–8]. CMR feature tracking (FT) is a recent magnetic resonance imaging tool that represents myocardial fibers deformation and also reflects early ventricular dysfunction [9, 10]. CMR-FT myocardial strain analysis has been studied in different heart diseases and was reported to have better prognostic information than ejection fraction [11]. Very few studies evaluated the regional analysis of left myocardial strain in different myocardial regions. They reported that the myocardial regions are not uniformly affected in β thalassemia patients with MIO [6]. Myocardial strain analysis can be used as a tool to identify high-risk patients who may need intensification of their chelation therapy and prioritized to get T2* CMR studies [1–7].

This study aims to assess the ability of CMR-FT left ventricular strain analysis for the early detection of systolic dysfunction in β thalassemia patients and to correlate it with the degree of MIO measured by CMR T2*.

## Methods

### Study population

This study is a prospective study conducted from January 2022 to December 2023. A total of 57 consecutive β thalassemia major patients received long-term blood transfusions (ranging from 1 to 4 packs/mo) were enrolled in the study. All study participants underwent CMR and CMR-FT. Among these 11 patients were diagnosed with MIO based on CMR T2* values. Patients with septal T2* less than 20 ms were considered to have MIO while others with septal T2* more than 20 ms were considered to be without MIO [12]. There were 46 patients (80.8%) without MIO and 11 patients (19.2%) with MIO. From the 11 patients with MIO, one had mild MIO (T2*, 15–20 ms), seven had moderate MIO (T2*, 10–15 ms) and three had severe MIO (T2*, < 10 ms). Twenty healthy subjects without any cardiac symptoms or previous diagnosis of cardiac condition were included as a control group. Exclusion criteria included patients with contraindications to magnetic resonance imaging, or patients suffering from another cardiac disease.

### CMR protocol

CMR was performed using a 1.5-T magnet (Philips Ingenia). Multiple slices during different phases of the cardiac cycle were obtained in the short-axis, axial, three and four-chamber planes by using free breathing retrospective electrocardiogram-gated steady-state free precession sequence. The following parameters were used: time of repetition, 3.20–3.65 ms; time to echo, 1.60–1.83 ms; field of view, 270 mm^2^; slice thickness, 5 mm; 30 cardiac phases; and no slice gap. T2* images were performed using black blood gradient echo sequence using time of repetition of 120 ms, different time to echo with 15 numbers of echoes, and flip angle of 20°. Signal intensity was measured in T2* images at different time to echo by a region of interest placed in the interventricular septum. Patients were categorized into two groups according to T2* values, patients with MIO when T2* < 20 ms and patients without MIO when T2* > 20 ms.

### CMR-FT interpretation

**Fig. 1:**
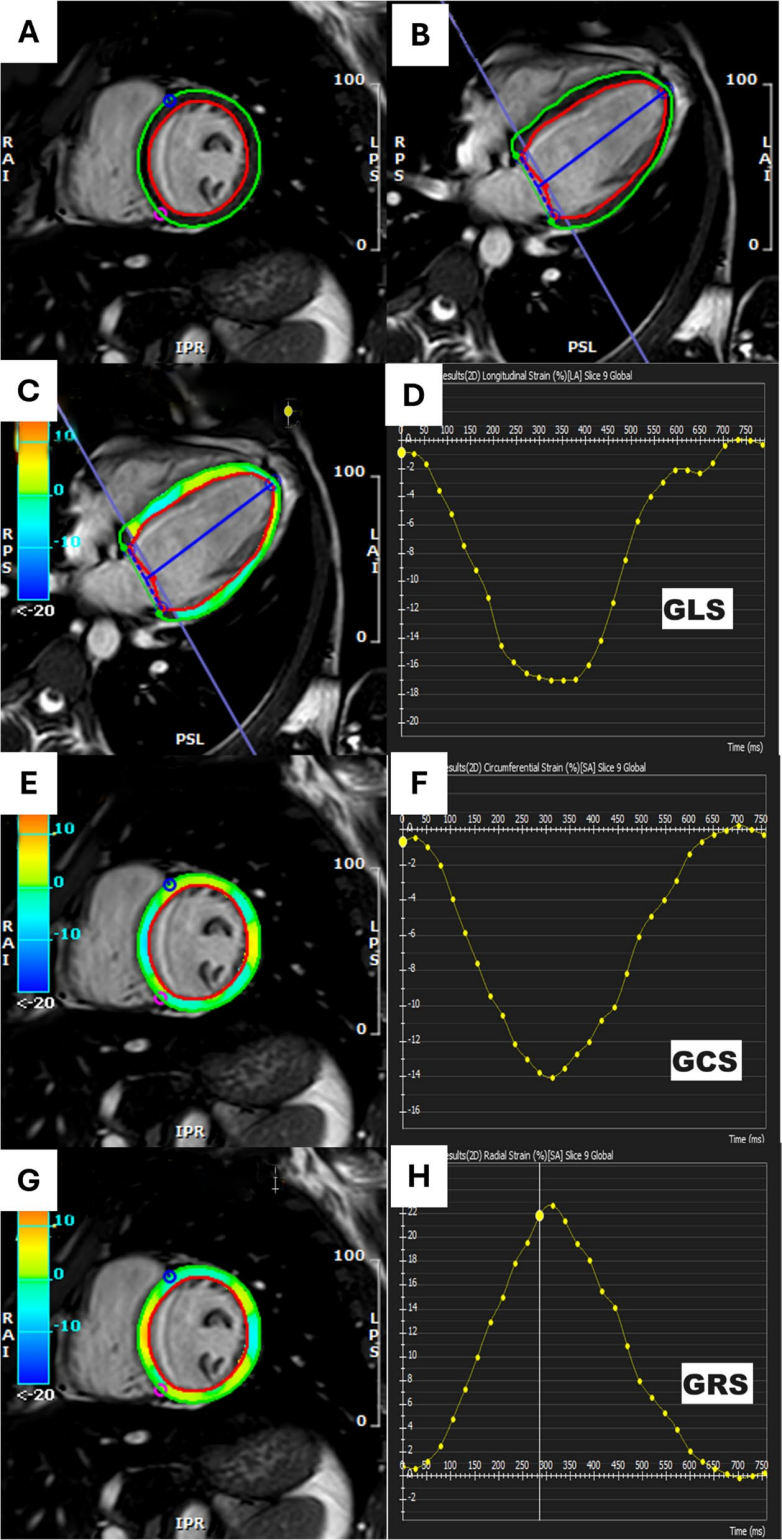
Feature traking analysis in a patient with β-thalassaemia major (**A**, **B**) Analysis of cardiac magnetic resonance imaging feature tracking strain of short-axis and four-chamber cine images. Color map and curve of (**C**, **D**) global longitudinal strain (GLS), (**E**, **F**) global circumferential strain (GCS), and (**G**, **H**) global radial strain (GRS) in a patient with thalassaemia major with preserved left ventricular ejection fraction

Two-dimensional (2D) FT analysis was performed offline using dedicated cardiac imaging software (CVI42, Circle Cardiovascular Imaging). Two radiologists with 10- and 7-years of experience in CMR performed the analysis in two separate reading sessions. They were blinded to the patient’s clinical data. Endocardial and epicardial contours of left ventricle (LV) were traced at the end-diastolic phase in short-axis and two-, three- and four-chamber planes. Automated tracking was visually reviewed, and the epicardial and endocardial contours were manually adjusted when required. The mean global longitudinal strain (GLS) analysis was performed using long-axis planes (two-, three- and four-chamber planes). The mean global radial strain (GRS) and global circumferential strain (GCS) analyses were performed using short-axis planes, as well as the regional strain analysis in the 16 myocardial segments. Global strain values, strain curves, strain rate curves, polar maps and cooler-coded images were obtained. Regional strain values were recorded for apical, mid, and basal regions (Fig. 1).

### Statistical analysis

Data were analyzed using IBM SPSS ver. 22.0 (IBM Corp). The Kolmogorov–Smirnov test was performed to determine the normality of the distribution of quantitative data. Quantitative data were described as mean standard deviation or median and range according to the normality test. The Mann–Whitney U-test and Student t-test were used to compare two groups for non-normally and normally distributed data, respectively. Interobserver agreement for strain analysis was evaluated in 40 β thalassemia major patients using intraclass correlation coefficients (ICCs) at 95% confidence intervals (CIs). The Pearson correlation test was used to correlate T2* with LV ejection fraction (EF) and global LV myocardial strain values. The one-way analysis of variance (ANOVA) test was performed to compare basal, mid, and apical strain values. Receiver operating characteristic (ROC) curve was performed to detect the cutoff value of GCS and GRS for differentiating β-thalassemia major patients from controls with the calculation of area under the curve (AUC), 95% CIs, sensitivity, and specificity. The chosen cutoff point was based on best detected cutoff point retrieved from the curve that yields highest sensitivity and specificity and for combined parameters; multivariate analysis was firstly done and then a new variable was detected by saved probability and from which we retrieve the best detected cutoff point. *P-*values were considered significant when they were less than 0.05.

## Results

### Patient demographics and clinical data

This study included 57 patients with β-thalassemia major (28 men [49.1%] and 29 women [50.9%]), with median age of 27 years (range, 18–50 years); and 20 healthy subjects (11 men [55.0%] and nine women [45.0%]) with median age of 29 years (range, 18–49 years) as the control group. Patients with β-thalassemia major were then divided into two groups according to T2* CMR results, 46 patients (80.8%) without MIO and 11 patients (19.2%) with MIO (one had mild MIO, seven had moderate MIO, and three had severe MIO).

The median serum ferritin in patients with MIO had trends to be higher than in patients without MIO (2,500 ng/mL vs. 1,766.5 ng/mL) but this difference did not reach statistical significance (*P* = 0.190). The median white blood cells, hemoglobin, platelets, liver enzymes (alanine transaminase and aspartate transferase), and albumin are displayed in Table 1, with no significant difference between both studied groups.

**Table 1:** Demographic criteria and clinical data of β-thalassemia major patients

Characteristic	Without MIO (*n* = 46)	With MIO (*n* = 11)	*P-*value
Age (yr)	30 (19–45)	24 (18–50)	0.041^*^
Ferritin (ng/mL)	1,766.5 (480–4,291)	2,500 (870–4,000)	0.190
White blood cell count (× 10^3^/uL)	23 (3.3–158)	22.4 (3.3–47)	0.867
Hemoglobin (g/dL)	7.8 (5.9–10.5)	7.5 (5.3–9.1)	0.125
Platelet count (× 10^3^/uL)	619 (101–1,985)	564 (26–941)	0.356
Alanine transaminase (IU/L)	32.5 (7–114)	48 (7–76)	0.598
Aspartate transferase (IU/L)	47.5 (15–144)	50 (16–121)	0.867
Albumin (gm/dL)	4.0 (1.4–4.9)	3.9 (1.7–4.9)	0.571

Values are presented as median (interquartile range). Mann–Whitney U-test was used to test significance

^*^P < 0.05

### CMR parameters

The mean LV EF was significantly lower in thalassemia patients compared to the control group (*P* = 0.003). Also, patients had higher mean LV end-diastolic volume index compared to the control group (101. mL/m^2^ vs. 75.6 mL/m^2^, *P* = 0.042). Comparison between different CMR parameters between thalassemia patients and control was demonstrated in Table 2.

**Table 2:** Comparison of cardiac magnetic resonance imaging parameters and LV global myocardial strain values in β-thalassemia major patients and control group

Parameter	β-Thalassemia major group (*n* = 57)	Control group (*n* = 20)	*P-*value
LV EF (%)	59.8 ± 4.7	65.00 ± 4.03	0.003^*^
LV EDVI (mL/m^2^)	101.0 ± 30.4	75.6 ± 11.3	0.042^*^
LV ESVI (mL/m^2^)	40.6 ± 13.8	28.0 ± 5.9	0.100
LV SV (mL)	102.8 ± 3.2	102.3 ± 14.9	0.953
LV EDWMI	44.2 ± 28.3	43.7 ± 9.8	0.965
LV GLS (%)	–17.8 ± 2.2	–18.3 ± 1.2	0.980
LV GCS (%)	–15.7 ± 2.0	–17.9 ± 1.3	0.002^*^
LV GRS (%)	25.5 ± 4.5	30.9 ± 4.4	0.006^*^

Values are presented as mean ± standard deviation. Student t-test was used to test significance

LV Left ventricle, EF Ejection fraction, *EDVI* End-diastolic volume index, *ESVI* End-systolic volume index, *SV* Stroke volume, *EDWMI* End-diastolic wall mass index, *GLS* Global longitudinal strain, *GCS* Global circumferential strain, *GRS* Global radial strain

^*^P < 0.05

### CMR-FT global strain parameters

**Fig. 2:**
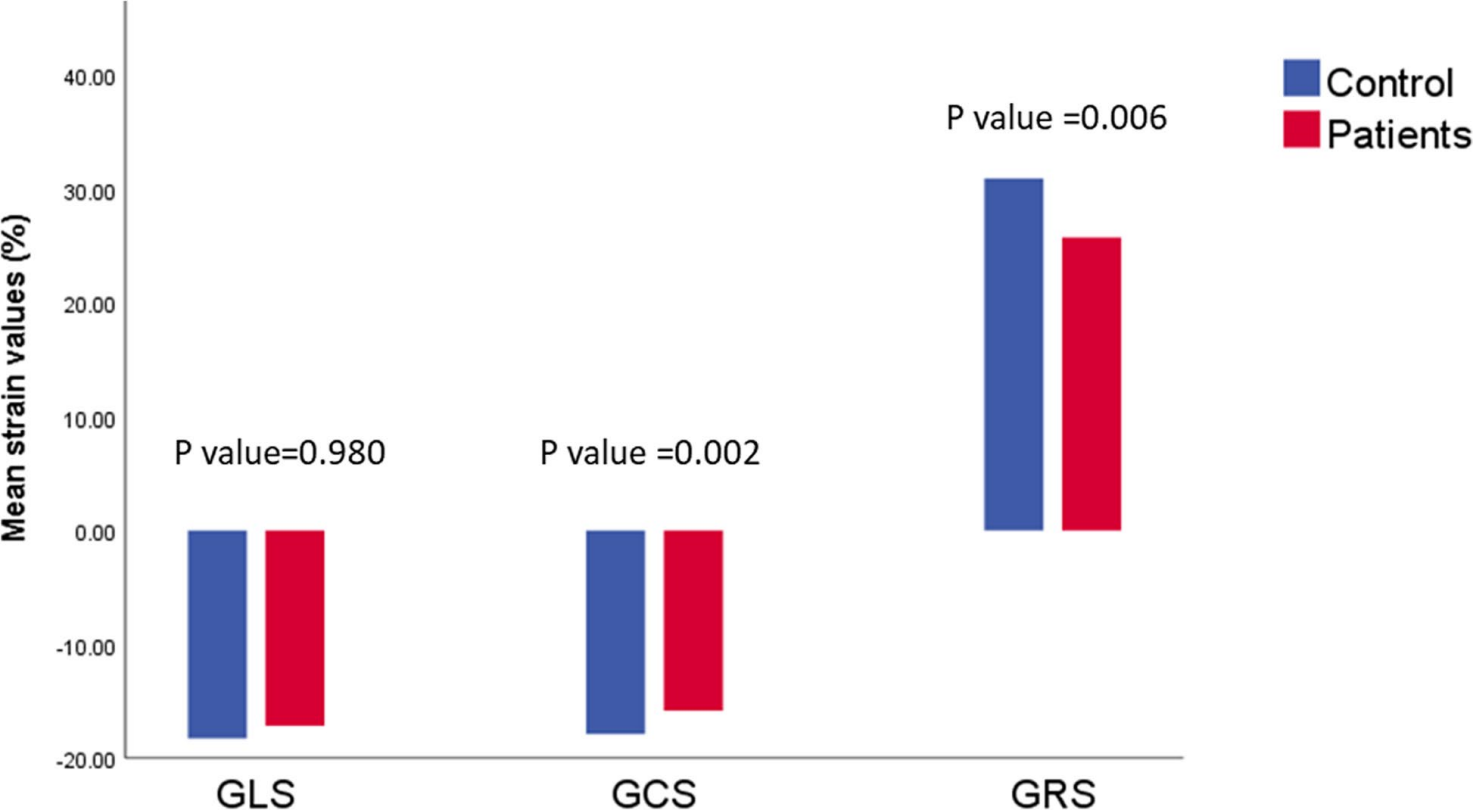
Bar chart showing statistically significant difference between β-thalassaemia major patients and control group regarding the mean left ventricle global longitudinal strain (GLS), global circumferential strain (GCS), and global radial strain (GRS)

**Fig. 3:**
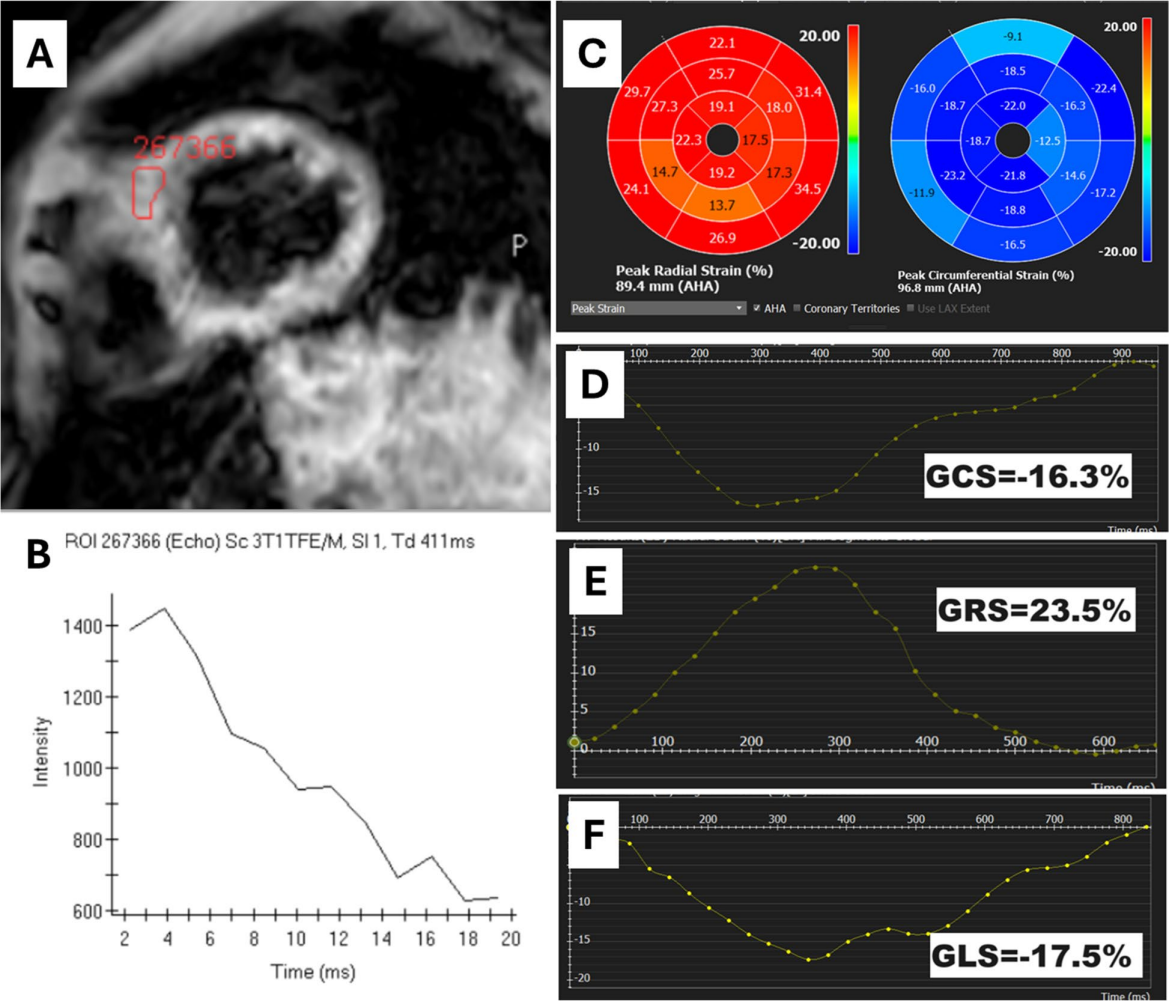
T2* and left ventricular strain analysis in a patient with β-thalassamia major and mild myocardial iron overload, (**A**) detected by T2* short-axis, (**B**) showing T2* of 19.3 ms and myocardial iron concentration of 1.21 mg/g. **C** Segmental global radial strain (GRS) and global circumferential strain (GCS). Strain curves for (**D**) GCS, (**E**) GRS, and (**F**) global longitudinal strain (GLS)

On comparing CMR-FT LV myocardial strain values between patients and control, the mean GCS and GRS values were significantly impaired in patients compared to the control group (*P* = 0.002 and *P* = 0.006, respectively). The mean GLS tends to be lower in patients compared to the control group, but did not reach statistical significance (*P* = 0.980) (Table 2, Fig. 2). There was no significant difference between patients with and without MIO as regards to GLS (*P* = 0.678), GCS (*P* = 0.572), and GRS (*P* = 0.771) (Table 3). An example of T2* and LV strain analysis in a patient with β-thalassemia major and mild myocardial iron overload is shown at Fig. 3.

**Table 3:** Comparison of cardiac magnetic resonance imaging parameters and LV global myocardial strain values in β-thalassemia major patients with and without MIO

Parameter	Without MIO (*n* = 46)	With MIO (*n* = 11)	*P-*value
LV EF (%)	60.0 ± 4.7	58.8 ± 4.8	0.423
LV EDVI (mL/m^2^)	109.9 ± 21.7	89.2 ± 15.5	0.007^*^
LV ESVI (mL/m^2^)	44.1 ± 11.4	36.2 ± 7.1	0.013^*^
LV SV (mL)	107.6 ± 22.8	83.9 ± 18.6	0.002^*^
LV EDWMI	44.1 ± 30.2	44.5 ± 22.0	0.966
LV EDWM to EDV ratio	0.4 ± 0.3	0.5 ± 0.2	0.224
T2* (msec)	38.4 ± 10.9	11.4 ± 3.4	< 0.001^*^
Myocardial iron concentration (mg/g)	0.6 ± 0.2	2.6 ± 0.9	< 0.001^*^
LV GLS (%)	–17.3 ± 2.2	–16.9 ± 2.0	0.678
LV GCS (%)	–15.8 ± 2.1	–16.1 ± 1.6	0.572
LV GRS (%)	25.6 ± 4.8	26.1 ± 3.4	0.771

Values are presented as mean ± standard deviation. Student t-test was used to test significance

*LV* Left ventricle, *MIO* Myocardial iron overload, *EF* Ejection fraction, *EDVI* End-diastolic volume index, *ESVI* End-systolic volume index, *SV* Stroke volume, *EDWMI* End-diastolic wall mass index, *EDWM* End-diastolic wall mass, *EDV* End-diastolic volume, *GLS* Global longitudinal strain, *GCS* Global circumferential strain, *GRS* Global radial strain

^*^P < 0.05

**Fig. 4:**
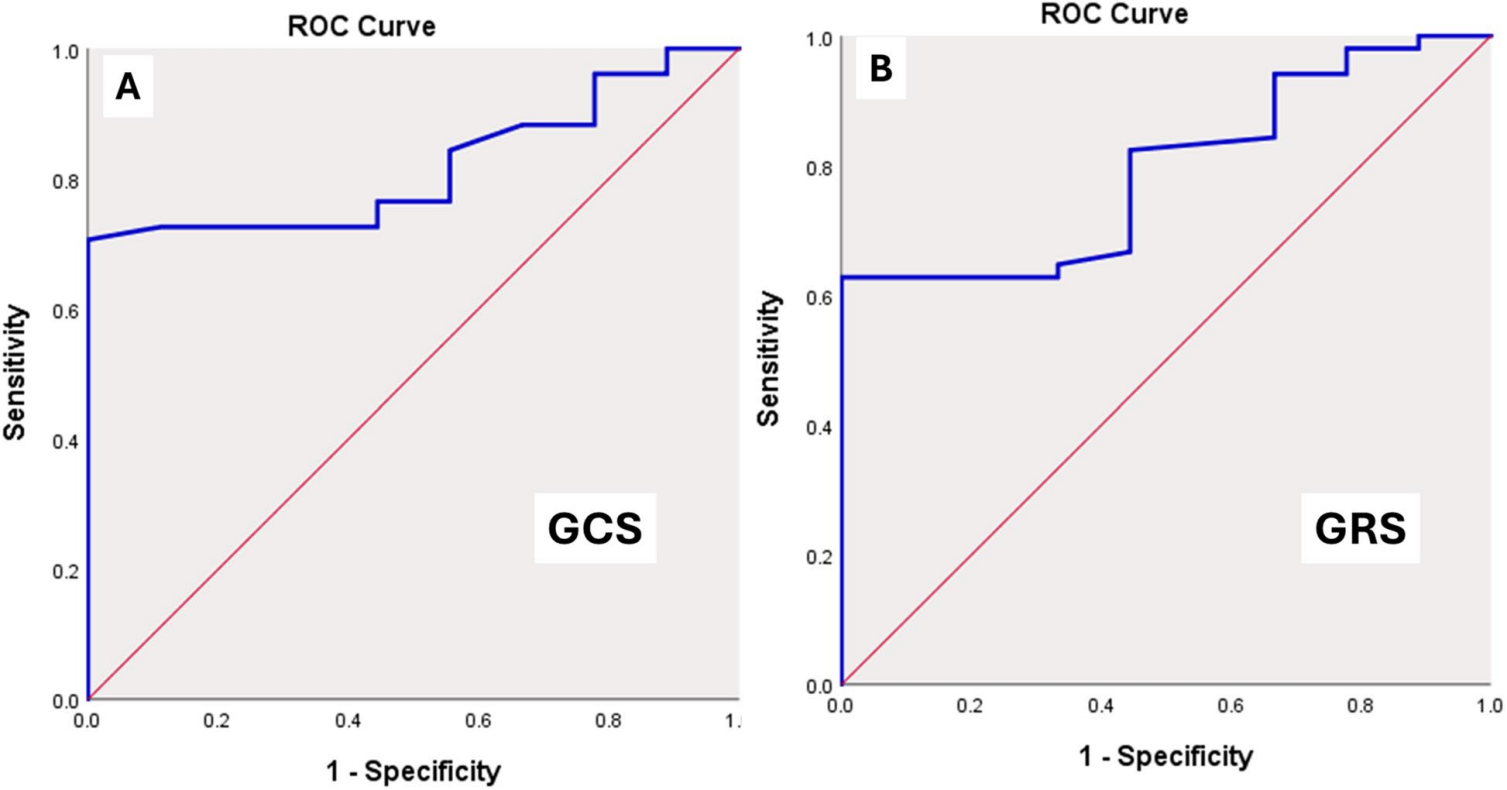
Receiver operating characteristic (ROC) curve analysis for differentiating β thalassaemia major patients from the control group. **A** ROC curve analysis of the left ventricle global circumferential strain (GCS) showing an area under the curve of 0.818 (*P* = 0.003). **B** ROC curve analysis of the left ventricle global radial strain (GRS) showing an area under the curve of 0.792 (*P* = 0.006)

ROC analysis revealed an AUC of 0.818 (95% CI, 0.709–0.928) with a GCS cutoff value of –16.95%, sensitivity of 72%, and specificity of 88% for GCS (*P* = 0.003). While for GRS, the cutoff value of 27.2% showed an AUC of 0.792 (95% CI, 0.664–0.920), sensitivity of 63%, and specificity of 78% (*P* = 0.006) (Fig. 4 A, B).

The mean GCS and GRS values were lower in β-thalassemia patients without MIO compared to the control group (*P* = 0.012 and *P* = 0.007, respectively). However, the mean GLS values did not show a significant difference between the two groups (*P* = 0.420) (Table 4).

**Table 4:** Comparison of cardiac magnetic resonance imaging parameters and LV global myocardial strain values in β-thalassemia major patients without MIO and control group

Parameter	Without MIO group (*n* = 46)	Control group (*n* = 20)	*P-*value
LV EF (%)	60.3 ± 4.75	65.0 ± 4.0	0.014^*^
LV EDVI (mL/m^2^)	106.6 ± 20.6	79.6 ± 11.3	0.000^*^
LV ESVI (mL/m^2^)	42.5 ± 10.8	28.0 ± 5.9	0.000^*^
LV SV (mL)	105.0 ± 19.8	102.3 ± 14.9	0.679
LV GLS (%)	–17.3 ± 2.3	–18.3 ± 1.2	0.420
LV GCS (%)	–15.8 ± 2.1	–17.9 ± 1.3	0.012^*^
LV GRS (%)	25.5 ± 4.8	30.9 ± 4.4	0.007^*^

Values are presented as mean ± standard deviation. Student t-test was used to test significance

*LV* Left ventricle, *MIO* Myocardial iron overload, *EF* Ejection fraction, *EDVI* End-diastolic volume index, *ESVI* End-systolic volume index, *SV* Stroke volume, *GLS* Global longitudinal strain, *GCS* Global circumferential strain, *GRS* Global radial strain

^*^P < 0.05

### CMR-FT regional strain parameters

LV regional myocardial strain values revealed statistically significant differences between the basal, mid and apical segments regarding GRS, GRS rate, GCS and GCS rate (*P* < 0.001 for all). Post hoc analysis in the one-way ANOVA test between regional myocardial strain values in the basal, mid, and apical regions revealed statistically significant differences between basal and apical regions concerning GRS, GRS rate, and GCS (*P* = 0.000 for all). There was a statistically significant difference between mid and apical regions concerning GRS (*P* = 0.014), GRS rate (*P* = 0.003), GCS (*P* = 0.050), and GCS rate (*P* = 0.001). GCS rate was significantly different between basal and mid regions (*P* = 0.001). However, GRS (*P* = 0.246), GRS rate (*P* = 0.621), and GCS (*P* = 0.090) did not show statistically significant differences between basal and mid regions. The mean base to apex ratio was 0.82% ± 0.27% for GRS and 0.86% ± 0.2% for GCS (Table 5).

**Table 5:** Comparison between different left ventricle myocardial regions concerning myocardial strain parameters

Parameter	Segment			*P-*value			
	Basal	Mid	Apical	Overall	Basal vs. mid	Basal vs. apical	Mid vs. apical
GRS (%)	25.7 ± 0.5	28.4 ± 5.9	34.0 ± 11.2	0.000^*^	0.246	0.000^*^	0.014^*^
GRS rate	1.4 ± 0.4	1.5 ± 0.4	1.9 ± 0.8	0.000^*^	0.621	0.000^*^	0.003^*^
GCS (%)	–15.8 ± 2.5	–17.2 ± 2.3	–18.9 ± 3.8	0.000^*^	0.090	0.000^*^	0.050^*^
GCS rate	1.0 ± 0.4	1.0 ± 0.3	1.4 ± 0.5	0.000^*^	0.001^*^	0.981	0.001^*^

Values are presented as mean standard deviation. One-way analysis of variance was used to test significance

*GRS* Global radial strain, *GCS* Global circumferential strain

^*^P < 0.05

### Interobserver reliability of CMR-FT parameters

Interobserver agreement for LV strain analysis showed moderate agreement for GLS (ICC, 0.7; 95% CI, 0.4–0.8) and good agreement for both GCS (ICC, 0.86; 95% CI, 0.7–0.9) and GRS (ICC, 0.85; 95% CI, 0.7–0.9).

### Correlations between T2* values with LV EF, global myocardial strain values, and base to apex ratio

**Fig. 5:**
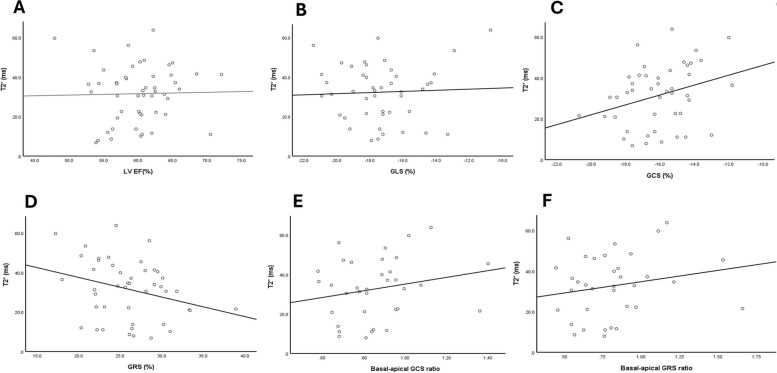
Correlation between T2* and (**A**) left ventricle (LV) ejection fraction (EF), (**B**) LV global longitudinal strain (GLS), (**C**) LV global circumferential strain (GCS), (**D**) LV global radial strain (GRS), (**E**) basal to apical LV GCS ratio, (**F**) basal to apical LV GRS ratio. Significant correlation was revealed between T2* and LV GCS (*r* = 0.361, *P* = 0.012) and LV GRS (*r* = –0.323, *P* = 0.025). However, no significant correlation was found between T2* and other parameters

In this study we found no correlation between T2* values and LV EF in thalassemia patients, (*r* = 0.23, *P* = 0.875). However, there was a significant positive correlation between T2* values and GCS values (*r* = 0.361, *P* = 0.012). We also found a significant negative correlation between T2* values and GRS values (*r* = –0.323, *P* = 0.025). GLS values did not significantly correlate with T2* values. There was no significant correlation between base to apex ratio and T2* values (*P* = 0.250 for GRS ratio and *P* = 0.225 for GCS ratio) (Fig. 5).

## Discussion

The main findings of this study are as follows. First, LV strain parameters (GCS and GRS) were significantly lower in thalassemia patients compared to control group. Second, thalassemia patients without MIO showed significantly lower LV GCS and LV GRS compared to control group. Third, there was significant correlation between LV GCS and LV GRS and T2* values even in absence of significant correlation with LV EF. Fourth, LV GCS and LV GRS values were lower in the basal and mid myocardial segments compared to the apical segments.

The intension from this study was not to replace the gold standard T2* used for detection of MIO but to detect the early impairment in LV function even in absence of MIO, as absence of MIO doesn’t mean that the myocardium is healthy, so the intension was to provide additional information from routine CMR sequences to detect early myocardial impairment.

Iron-induced cardiomyopathy is the main cause of death in thalassemia patients with chronic blood transfusion. Initially, excess iron saturates the myocardial iron storage in its bounded form. During this period, myocardial T2* value decreases, yet cardiac functions generally remain within normal limits. If myocardial iron is not cleared at this stage, iron exceeds the storage capacity of the myocardium, and free iron appears, which is more toxic to the myocardial cells and results in impaired myocardial function [12, 13]. Although, it appears that the main pathophysiologic event is related to iron overload, other physiologic, biomolecular, and structural factors play a role in affecting the myocardium [14].

Conventional echo cardiography has failed to identify the early myocardial dysfunction among β-thalassemia major patients, thus speckle tracking echocardiography (STE) has been lately used to detect early myocardial deformation [15]. We noted heterogeneity in the methods that were used in previous studies for evaluation of STE-derived LV strain parameters in β-thalassemia major patients, as some studies used 2D STE [15–28],while others used 3D STE [23, 29–31]. Regarding 2D-STE for LV GLS estimation, some studies acquired it from single apical four-chamber view [25, 28, 29], others acquired it from the combination of apical four- and two-chamber views [27, 32, 33], while in the remaining studies it was acquired from the combined apical four-, three-, and two-chamber views [16, 18–22, 24, 34, 35]. All previously mentioned studies agreed on using parasternal short-axis view for acquiring 2D-STE–derived GCS and GRS indices [16, 21, 26–29, 31, 32]. This technical difference can explain the different results among the literature. As, when comparing patient with controls, some studies reported significant difference between the two groups regarding GLS [18, 19, 29, 30, 34], GCS [16, 29, 31] or GRS [27]. While others reported no significant difference between the two groups regarding GLS [16, 24, 25], GCS [26], or GRS [28]. In addition, some of the previously mentioned studies reported significant difference between cases with MIO and cases without MIO regarding GLS or GCS [35], However others found no significant difference between the two groups regarding GLS [36], GCS [16, 20, 29], or GRS [16, 29].

Recently, CMR-FT has been used for detection of myocardial deformation. CMR has the privilege of high contrast and spatial resolution, multiplanar imaging and being nonoperator dependent unlike echo. Regarding CMR-FT parameters, previous studies agreed on the method by which they acquire these parameters. As they obtained GLS using four-chamber with or without two- and three-chamber views while GCS and GRS were acquired using short-axis view [1, 2, 14, 15]. Many previous studies agreed that LV strain is impaired in thalassemia patients, however, there were controversies on the best strain parameter that can detect the early impairment in cardiac function [1, 2, 14, 15]. Previous studies on CMR and autopsy findings revealed that in thalassemia patients, the subepicardial layer of the myocardium is the main site of iron deposition [15]. The subepicardial layer is the most important marker of cardiac rotation [37]. It was reported that the deformation of the subepicardial layer is reflected as impaired LV GRS [37] and LV GCS [1]. This can explain our findings that the mean GCS and GRS values were significantly lower in thalassemia patients compared to the control group.

LV GLS reflects the longitudinal shortening of the subendocardial layer [1, 11]. Since MIO begins in the subepicardial layer, it is expected that GLS may be spared till late stages of the disease [1]. This fact can explain our results that there was no significant difference between patients and control group regarding GLS values. However, some previous studies reported that GLS was the most valuable strain parameter in detecting early LV dysfunction in thalassemia patients. However, these studies did not reach an explanation for this finding, and they did not correlate the GLS values with the T2* values [8, 38].

Unlike previously mentioned studies, there has been a great debate regarding correlation between LV myocardial strain, either obtained by STE or by CMR, and T2* values and so the degree of MIO. Most of the previous studies that used STE to evaluate the LV strain in thalassemia patients reported a significant correlation between GLS and T2* [8, 20, 35, 38–40], only two studies reported significant correlation between GCS and T2* [20, 35], yet none of the previously mentioned studies found significant correlation between GRS and T2* (Table 6 [8, 20, 35, 38–40]). Regarding strain parameters derived from CMR, three previous studies reported significant correlation between GRS and T2* [1, 3, 37], one study reported a significant correlation between GCS and T2* [13], and one study significant correlation between GLS and T2* (Table 6 [37]). This variability among previously reported results can be explained by the hypothesis that not only MIO is the cause of myocardial damage in thalassemia patients. Those patients suffer from chronic anemia which can cause chronic hypoxia explaining the affection of LV subendocardial layer and so reduction of GLS regardless of the degree of iron overload as mentioned in some of previous studies [1]. Thalassemia patients also receive repeated blood transfusion among their life increasing the possibility of having viral infection such as hepatitis C. Also, various degrees of genetic factors, and chronic state of immunodeficiency in these patients can contribute in myocardial damage [14]. This explains why the relation between strain values (reflecting LV function) and T2* (reflecting degree of MIO) is not linear and not constant among studies. So, although early detection of MIO is important, absence of MIO does not exclude myocardial affection and hence those patients require surveillance of their cardiac function for early detection of myocardial impairment allowing them to receive adequate treatment as early as possible. In agreement with some of the previous studies, we found a significant correlation between both LV myocardial strain parameters (GRS and GCS) and T2* values in thalassemia patients despite the absence of a significant correlation between EF and T2* values. Accordingly, this supports the potential of using CMR strain analysis for the early detection of subtle systolic dysfunction in thalassemia patients before it can be detected by conventional functional CMR even in absence of significant MIO.

**Table 6:** Literature review showing correlation between T2* and the three LV strain parameters

Study	No. of thalassemia patients	Method of strain acquisition	T2*	GLS	GCS	GRS	T2* correlation with GLS		T2* correlation with GCS		T2* correlation with GRS	
							r	*P-*value	r	*P-*value	r	*P-*value
Garceau et al. [35] (2011)	45	2D-STE: LV GLS (A4C, A3C, A2C), LV GCS (PSAX)	23 ± 13	–18.0 ± 3.0	–22.0 ± 5.0	-	–0.65	< 0.05^*^	–0.39	< 0.05^*^	-	-
Cusmà Piccione et al. [38] (2013)	32	2D-STE: LV GLS (A4C, A2C), LV GRS, LV GCS (PSAX)	37.7 ± 5.5	–17.9 ± 3.5^*^	–20.5 ± 5.1	36.7 ± 8.2	–0.53	0.001^*^	-	-	-	-
Li et al. [39] (2016)	24	3D-STE: LV GLS	32.7 ± 16.7	28.0 ± 7.4^*^	-	-	0.74	< 0.001^*^	-	-	-	-
Pizzino et al. [8] (2018)	28	2D-STE: LV GLS (A4C, A3C, A2C), LV GCS (PSAX)	40.5 (32 to 44)	–20.6 ± 2.8	–19.5 ± 2.6	–18.3 ± 2.0^*^	–0.41	0.031^*^	-	-	-	-
Poorzand et al. [42] (2017)	44	2D-STE: LV GLS (A4C, A3C, A2C), LASr, RASr (A4C)	19.8 ± 10.3	–19.4 ± 3.2	-	–17.6 ± 2.6^*^	–0.42	0.001^*^	-	-	-	-
Abtahi et al. [40] (2019)	52	2D-STE: LV GLS (A4C, A3C, A2C)	19.8 ± 10.3	–19.4 ± 3.2	–17.6 ± 2.6^*^	–21.6 ± 2.7	–0.6	0.001^*^	-	-	-	-
Fattahi et al. [20] (2021)	48	2D-STE: LV GLS (A4C, A3C, A2C) 3D-STE: LV GLS, LVGCS	24.2 (3 to 43)	–22.0 ± 4.6	–30.1 ± 6.5	–21.6	–0.5^a^	< 0.05^*^	–0.49^b^	< 0.05^*^	-	-
Alis et al. [13] (2022)	42	CMR-FT	30.97 ± 10.84	16.85 ± 1.73	16.86 ± 1.96	28.25 ± 4.79	-	-	0.6	< 0.0001^*^	-	
Asadian et al. [37] (2021)	154	CMR-FT	24.77 ± 11.18	18.07 ± 1.35	17.68 ± 1.78	39.82 ± 7.77	0.309	< 0.001^*^	-	-	0.368	< 0.001^*^
Ojha et al. [1] (2021)	104	CMR-FT	31.37 (24.37 to 39.08)	− 14.53 ± 2.86	–19.75 (–22.02 to –18.06)	33.41 (27.58 to 41.09)	–0.17	0.10	–0.16	0.12	0.24	0.02^*^
Das et al. [3] (2022)	89	CMR-FT	-	–16.8 ± 3.80	–18.2 ± 3.44	46.76 ± 9.74	–0.194	0.085	–0.217	0.053	0.232	0.038

Values are presented as mean ± standard deviation or median (range)

*LV* Left ventricle, *GLS* Global longitudinal strain, *GCS* Global circumferential strain, *GRS* Global radial strain, *D* Dimensional, *STE* Speckle tracking echocardiography, *A4C* Apical four-chamber, *A3C* Apical three-chamber, *A2C* Apical two-chamber, *PSAX* Parasternal short-axis view, *LASr* Left atrial strain at reservoir phase, *RASr* Right atrial strain at reservoir phase, *CMR* Cardiac magnetic resonance imaging, *FT* Feature tracking

^a^3D-STE LV GLS. ^b^3D-STE LV GCS

^*^P < 0.05

The results regarding the LV CMR-FT parameters between β-thalassemia major patients with and without MIO are controversial. While some studies reported that only GRS values were significantly different between the two groups [1], others found a significant difference in GCS [2, 14, 15] and GLS [2]. Odoardo et al. [32], used STE strain analysis to assess myocardial strains in 55 thalassemia patients and they reported significant difference regarding GCS and GRS between patients and control; however, they found no significant difference regarding the three strain parameters between thalassemia patients with and without MIO. In agreement with the previously mentioned study, we found no statistically significant difference in GLS and GCS values between patients with MIO and patients without MIO. However, GRS was slightly lower in patients with MIO compared to those without MIO, but this difference did not reach statistical significance. This finding supports the claim that the relationship between MIO and myocardial dysfunction is not linear as there are other factors contributing factors to the myocardial damage other than MIO. This also might be explained by the smaller number of patients with MIO enrolled in our study.

It was reported that some thalassemia patients with MIO, especially those with higher levels of serum ferritin, develop restrictive LV filling in response to MIO [41]. This may be the explanation of our findings as we noted that end-diastolic volume index and end-systolic volume index were significantly lower in thalassemia patients with MIO compared to those without MIO, although there was no significant difference regarding the end-diastolic wall mass index among the two groups. Also, the serum ferritin was higher in cases with MIO compared to those without MIO although this difference did not reach a statistical significance.

Alis et al. [13] found in their study that thalassemia patients without MIO (with T2* > 20 ms) showed significantly lower GCS and GRS values compared to the control group, but they found no significant difference between the two groups regarding GLS. On the other hand, Asadian et al. [37] found that GLS was the only strain parameter that was different between the two groups. In our study, both GCS and GRS were lower in thalassemia patients without MIO compared to the control group. This finding can be explained by the claim that the affection of LV myocardium in those patients might be multifactorial including inflammatory, molecular, and immunologic factors and not only secondary to MIO [14].

Segmental strain analysis in this study revealed significantly lower LV strain values (GRS and GCS) in the basal and mid myocardial segments compared to the apical segments, suggesting the possibility of sparing the apical segments until late in the disease. These results were in line with Rao et al. [7] who reported a statistically significant difference between the apical and basal segments for both longitudinal and circumferential strains. This might be explained by the hypothesis that iron deposition early in the disease is not uniform throughout the myocardium and tends to spar apical segments [7, 16].

This study has several limitations. First, the small number of patients especially the MIO group (11 cases), further research on a larger number of patients is needed for a better understanding of the role of CMR strain analysis in thalassemia patients. Second, this cross-sectional study showed impaired LV strain values in thalassemia cases, we did not perform future follow-up for cardiac events. Longitudinal studies are recommended to evaluate the prognostic role of CMR strain for assessment of systolic function. Third, the study was executed in a single-center arrangement with a single-vendor application, further multicenter studies are needed to establish the role of strain analysis in thalassemia patients.

## Conclusions

β-Thalassemia major patients can have myocardial dysfunction even in absence of MIO. CMR-FT strain parameters can play an important role as a marker for the early detection of systolic impairment in those patients. The correlation of myocardial strain parameters with T2* values may help in the identification of high-risk groups who may need modification of the dose of their iron chelation therapy.

## Data Availability

All data generated or analyzed during this study are included in this published article.

## Abbreviations

ANOVA: Analysis of variance
AUC: Area under the curve
CI: Confidence interval
CMR: Cardiac magnetic resonance imaging
D: Dimensional
EF: Ejection fraction
FT: Feature tracking
GCS: Global circumferential strain
GLS: Global longitudinal strain
GRS: Global radial strain
ICC: Intraclass correlation coefficient
LV: Left ventricle
MIO: Myocardial iron overload
ROC: Receiver operating characteristic
STE: Speckle tracking echocardiography

## References

[1] Ojha V, Ganga KP, Seth T, Roy A, Naik N, Jagia P, et al. Role of CMR feature-tracking derived left ventricular strain in predicting myocardial iron overload and assessing myocardial contractile dysfunction in patients with thalassemia major. Eur Radiol. 2021;31:6184–92. 33721061 10.1007/s00330-020-07599-7

[2] Rezaeian N, Asadian S, Parsaee M, Toloueitabar Y, Hemmati Komasi MM, Shayan L, et al. The predictive role of cardiac magnetic resonance imaging in determining thalassemia patients with intermediately to highly probable pulmonary hypertension. Echocardiography. 2021;38:1769–77. 34596897 10.1111/echo.15210

[3] Das KM, Baskaki UM, Pulinchani A, Ali HM, Almanssori TM, Gorkom KV, et al. Significance of cardiac magnetic resonance feature tracking of the right ventricle in predicting subclinical dysfunction in patients with thalassemia major. Diagnostics (Basel). 2022;12:1920. 36010270 10.3390/diagnostics12081920 PMC9406855

[4] Batouty NM, Sobh DM, Tawfik AM. Native myocardial T1 mapping in β-thalassemia major patients with and without iron overload. Acta Haematol Pol. 2023;54:48–9. 10.5603/AHP.a2023.0008

[5] Ramazzotti A, Pepe A, Positano V, Rossi G, De Marchi D, Brizi MG, et al. Multicenter validation of the magnetic resonance T2* technique for segmental and global quantification of myocardial iron. J Magn Reson Imaging. 2009;30:62–8. 19557847 10.1002/jmri.21781

[6] Hamdy AM. Use of strain and tissue velocity imaging for early detection of regional myocardial dysfunction in patients with beta thalassemia. Eur J Echocardiogr. 2007;8:102–9. 16564231 10.1016/j.euje.2006.02.004

[7] Rao S, Hasan BS, Hoodbhoy Z, Habib I, Mohsin S, Tomredle R, et al. Role of myocardial deformation imaging in transfusion-dependent thalassemia: correlation with severity of myocardial siderosis. Prog Pediatr Cardiol. 2023;68: 101607. 10.1016/j.ppedcard.2022.101607

[8] Pizzino F, Meloni A, Terrizzi A, Casini T, Spasiano A, Cosmi C, et al. Detection of myocardial iron overload by two-dimensional speckle tracking in patients with beta-thalassaemia major: a combined echocardiographic and T2* segmental CMR study. Int J Cardiovasc Imaging. 2018;34:263–71. 28770456 10.1007/s10554-017-1219-7

[9] Chitiboi T, Axel L. Magnetic resonance imaging of myocardial strain: a review of current approaches. J Magn Reson Imaging. 2017;46:1263–80. 28471530 10.1002/jmri.25718

[10] Tawfik AM, Sobh DM, Gadelhak B, Zedan MM, Sobh HM, Eid R, et al. Right ventricular strain analysis by tissue tracking cardiac magnetic resonance imaging in pediatric patients with end-stage renal disease. J Thorac Imaging. 2024;39:49–56. 37265246 10.1097/RTI.0000000000000716

[11] Sobh DM, Batouty NM, Tawfik AM, Gadelhak B, Elmokadem AH, Hammad A, et al. Left Ventricular strain analysis by tissue tracking-cardiac magnetic resonance for early detection of cardiac dysfunction in children with end-stage renal disease. J Magn Reson Imaging. 2021;54:1476–85. 34037288 10.1002/jmri.27700

[12] Anderson LJ, Holden S, Davis B, Prescott E, Charrier CC, Bunce NH, et al. Cardiovascular T2-star (T2*) magnetic resonance for the early diagnosis of myocardial iron overload. Eur Heart J. 2001;22:2171–9. 11913479 10.1053/euhj.2001.2822

[13] Alis D, Asmakutlu O, Topel C, Sahin AA, Karaarslan E. Association between left ventricular strain and cardiac iron load in beta-thalassaemia major: a cardiac magnetic resonance study. Acta Cardiol. 2022;77:71–80. 33685353 10.1080/00015385.2021.1887585

[14] Gujja P, Rosing DR, Tripodi DJ, Shizukuda Y. Iron overload cardiomyopathy: better understanding of an increasing disorder. J Am Coll Cardiol. 2010;56:1001–12. 20846597 10.1016/j.jacc.2010.03.083 PMC2947953

[15] Patsourakos D, Aggeli C, Dimitroglou Y, Delicou S, Xydaki K, Koukos M, et al. Speckle tracking echocardiography and β-thalassemia major: a systematic review. Ann Hematol. 2023 Aug 1 [Epub]. 10.1007/s00277-023-05380-6 10.1007/s00277-023-05380-6 PMC11358224 37526674

[16] El-Shanshory M, Tolba O, El-Shafiey R, Elgamasy M, Hablas N, Mawlana W. Cardiac iron overload by MRI in children with B-thalassemia major and its correlation with cardiac function by echocardiography. J Pediatr Hematol Oncol. 2020;42:398–402. 32251156 10.1097/MPH.0000000000001786

[17] AbdelMassih AF, Salama KM, Ghobrial C, Haroun B, Rahman MA. Discrepancy in patterns of myocardial involvement in beta-thalassaemia vs. sickle cell anaemia. Acta Cardiol. 2020;75:442–9. 31165673 10.1080/00015385.2019.1610836

[18] Nashat M, Khedr LA, Khairat E, Elsheikh E. Evaluation of right and left ventricular function using speckle-tracking echocardiography in thalassemic patients. Ann Pediatr Cardiol. 2021;14:476–84. 35527770 10.4103/apc.apc_162_19 PMC9075552

[19] Sayed NM, Mashahit MA, El-Husseiny NM, Ali RA, Ahmed GA, Ibrahim MK. Cardiac structural and functional changes evaluated by echocardiography and two-dimensional strain in patients with beta thalassemia. Nat Sci. 2021;19:61–6.

[20] Fattahi H, Parsaee M, Rezaeian N, Azarkeivan A, Meimand SE, Mohammadi K, et al. Comparison between two and three-dimensional speckle-tracking echocardiography and cardiac T2* magnetic resonance imaging in ß-thalassemia. Res Cardiovasc Med. 2021;10:7–13. 10.4103/rcm.rcm_15_21

[21] Cheung YF, Liang XC, Chan GC, Wong SJ, Ha SY. Myocardial deformation in patients with Beta-thalassemia major: a speckle tracking echocardiographic study. Echocardiography. 2010;27:253–9. 20070362 10.1111/j.1540-8175.2009.01005.x

[22] Barbero U, Fornari F, Gagliardi M, Fava A, Giorgi M, Alunni G, et al. Myocardial longitudinal strain as the first herald of cardiac impairment in very early iron overload state: an echocardiography and biosusceptometry study on beta-thalassemia patients. Am J Cardiovasc Dis. 2021;11:555–63. 34849287 PMC8611262

[23] Hanneman K, Nguyen ET, Thavendiranathan P, Ward R, Greiser A, Jolly MP, et al. Quantification of myocardial extracellular volume fraction with cardiac MR imaging in thalassemia major. Radiology. 2016;279:720–30. 26653680 10.1148/radiol.2015150341

[24] Okay M, Coteli C, Unal S, Hazırolan T, Karabulut E, Ozer N, et al. Strain imaging by speckle tracking for the assessment of diastolic dysfunction in beta-thalassemia major patients. Acta Medica. 2021;52:57–61.

[25] Cheung YF, So EK, Hwang GY, Chan GC, Ha SY. Left and right atrial function and remodeling in beta-thalassaemia major. Pediatr Cardiol. 2019;40:1001–8. 30972436 10.1007/s00246-019-02105-3

[26] Parsaee M, Saedi S, Joghataei P, Azarkeivan A, Alizadeh SZ. Value of speckle tracking echocardiography for detection of clinically silent left ventricular dysfunction in patients with β-thalassemia. Hematology. 2017;22:554–8. 28399703 10.1080/10245332.2017.1312206

[27] Chen MR, Ko HS, Chao TF, Liu HC, Kuo JY, Bulwer BE, et al. Relation of myocardial systolic mechanics to serum ferritin level as a prognosticator in thalassemia patients undergoing repeated transfusion. Echocardiography. 2015;32:79–88. 24673419 10.1111/echo.12590

[28] Karamanou AG, Hamodraka ES, Vrakas SC, Paraskevaidis I, Lekakis I, Kremastinos DT. Assessment of left ventricular and atrial diastolic function using two-dimensional (2D) strain imaging in patients with β-thalassemia major. Eur J Haematol. 2014;92:59–65. 24118422 10.1111/ejh.12209

[29] Eroğlu AG, Uluğ N, Karakaş H, Yüksel EK, Akyel NG, Çığ G, et al. Evaluation of left ventricular function and myocardial deformation in children with beta-thalassemia major by real-time three-dimensional (four-dimensional) and speckle tracking echocardiography. Echocardiography. 2022;39:1307–15. 36126339 10.1111/echo.15453

[30] Patsourakos D, Aggeli C, Gatzoulis KA, Delicou S, Dimitroglou Y, Xydaki K, et al. Left atrial deformation indices in β-thalassemia major patients. Ann Hematol. 2022;101:1473–83. 35460387 10.1007/s00277-022-04842-7

[31] El Razaky OA, El-Shanshory MR, El-Shehaby WA, Hables NM, Elshamia AM, Fayed AM, et al. Left ventricular regional function in children with beta thalassemia with no cardiac manifestations (four-dimensional echocardiographic study). Indian J Hematol Blood Transfus. 2019;35:750–7. 31741632 10.1007/s12288-019-01117-6 PMC6825061

[32] Di Odoardo LA, Giuditta M, Cassinerio E, Roghi A, Pedrotti P, Vicenzi M, et al. Myocardial deformation in iron overload cardiomyopathy: speckle tracking imaging in a beta-thalassemia major population. Intern Emerg Med. 2017;12:799–809. 28456904 10.1007/s11739-017-1670-4

[33] Ari ME, Ekici F, Çetin İİ, Tavil EB, Yaralı N, Işık P, et al. Assessment of left ventricular functions and myocardial iron load with tissue Doppler and speckle tracking echocardiography and T2* MRI in patients with β-thalassemia major. Echocardiography. 2017;34:383–9. 28139073 10.1111/echo.13463

[34] El-Shanshory M, Tolba O, El-Shafiey R, Mawlana W, Ibrahim M, El-Gamasy M. Cardioprotective effects of spirulina therapy in children with beta-thalassemia major. J Pediatr Hematol Oncol. 2019;41:202–6. 30531602 10.1097/MPH.0000000000001380

[35] Garceau P, Nguyen ET, Carasso S, Ross H, Pendergrast J, Moravsky G, et al. Quantification of myocardial iron deposition by two-dimensional speckle tracking in patients with β-thalassaemia major and Blackfan-Diamond anaemia. Heart. 2011;97:388–93. 21296782 10.1136/hrt.2010.192641

[36] Wood JC. Estimating tissue iron burden: current status and future prospects. Br J Haematol. 2015;170:15–28. 25765344 10.1111/bjh.13374 PMC4484399

[37] Asadian S, Rezaeian N, Hosseini L, Toloueitabar Y, Komasi MM, Shayan L. How does iron deposition modify the myocardium?: a feature-tracking cardiac magnetic resonance study. Int J Cardiovasc Imaging. 2021;37:3269–77. 34105082 10.1007/s10554-021-02305-0

[38] Cusmà Piccione M, Piraino B, Zito C, Khandheria BK, Di Bella G, De Gregorio C, et al. Early identification of cardiovascular involvement in patients with β-thalassemia major. Am J Cardiol. 2013;112:1246–51. 23871677 10.1016/j.amjcard.2013.05.080

[39] Li SJ, Hwang YY, Ha SY, Chan GC, Mok AS, Wong SJ, et al. Role of three-dimensional speckle tracking echocardiography in the quantification of myocardial iron overload in patients with beta-thalassemia major. Echocardiography. 2016;33:1361–7. 27158922 10.1111/echo.13266

[40] Abtahi F, Abdi A, Jamshidi S, Karimi M, Babaei-Beigi MA, Attar A. Global longitudinal strain as an Indicator of cardiac Iron overload in thalassemia patients. Cardiovasc Ultrasound. 2019;17:24. 31684963 10.1186/s12947-019-0174-y PMC6829819

[41] Kremastinos DT, Farmakis D. Iron overload cardiomyopathy in clinical practice. Circulation. 2011;124:2253–63. 22083147 10.1161/CIRCULATIONAHA.111.050773

